# Segment Anything Model for Gastric Cancer

**DOI:** 10.1002/cam4.71246

**Published:** 2025-09-23

**Authors:** Lanlan Li, Chongyang Wang, Yi Geng, Tao Chen, Ziyue Wang, Kaixin Lin, Hongan Wei, Jianping Wang, Dabiao Wang, Decao Niu, Juan Li

**Affiliations:** ^1^ College of Physics and Information Engineering Fuzhou University Fuzhou China; ^2^ Department of General Surgery (Colorectal Surgery) The Sixth Affiliated Hospital, Sun Yat‐sen University Guangzhou China; ^3^ Department of Gastroenterology Third People's Hospital, Fujian University of Traditional Chinese Medicine Fuzhou China; ^4^ College of Chemical and Engineering Fuzhou University Fuzhou China; ^5^ Department of Urology Guangdong Second Provincial General Hospital Guangzhou China; ^6^ Department of Endoscopic Surgery The Sixth Affiliated Hospital, Sun Yat‐sen University Guangzhou China; ^7^ Department of General Surgery (Endoscopic Surgery) The Sixth Affiliated Hospital, Sun Yat‐sen University Guangzhou China; ^8^ The Guangdong Provincial Key Laboratory of Colorectal and Pelvic Floor Diseases The Sixth Affiliated Hospital, Sun Yat‐sen University Guangzhou China; ^9^ The Biomedical Innovation Center The Sixth Affiliated Hospital, Sun Yat‐sen University Guangzhou China

**Keywords:** fine‐tune, gastric cancer, image segmentation, knowledge distillation, SAM

## Abstract

**Background:**

Gastric cancer is a biologically aggressive disease, accounting for a substantial proportion of cancer‐related deaths globally. Accurate localization of the lesion by artificial intelligence techniques helps timely and efficiently diagnose and treat. Segment Anything Model (SAM) has demonstrated considerable potential in medical image segmentation by displaying high performance in numerous image benchmark tests. However, its resource‐intensive nature limits feasibility in embedded medical contexts.

**Methods:**

This study proposed GC‐SAM, a lightweight model for tumor segmentation. The architecture of GC‐SAM is innovatively proposed, including a knowledge distillation image encoder, prompt encoder, and mask decoder, which effectively replaces the conventional fixed and computationally intensive network components.

**Results:**

Extensive experiments demonstrate that GC‐SAM significantly outperforms both classical segmentation models and recent state‐of‐the‐art networks. On the internal test set, GC‐SAM achieves 0.8186 Dice and 0.6504 mIoU, while reducing inference time and parameter count by over 80% compared to the original SAM. On the external dataset, GC‐SAM maintains superior performance (Dice 0.8350), demonstrating excellent generalization.

**Conclusions:**

The proposed GC‐SAM model shows strong capability in segmenting gastric cancer tissue, while also demonstrating practical potential for deployment in embedded medical imaging devices.

## 1 Introduction

Gastric cancer is recognized as a major health concern worldwide due to its elevated incidence and mortality. The disease significantly shortens patient survival, thereby contributing to a substantial global healthcare burden. The relevant examinations for gastric cancer include Upper Gastrointestinal Series (UGI), Gastroscopy, Computed Tomography (CT), Positron Emission Tomography‐Computed Tomography (PET‐CT), Endoscopic Ultrasonography (EUS) and Pathology [1]. Among these, histopathology diagnosis [2, 3] is considered the gold standard in gastric cancer diagnosis. To diagnose gastric cancer pathologically, professional pathologists must examine the morphologies and arrangement of cells in pathological images from histopathological slides [4]. However, manual diagnosis by pathologists is time‐consuming and subjective, easily influenced by their own experiences, highlighting the need for continuous exploration of alternative approaches to improve efficiency and reliability.

Medical image segmentation has become a prominent research focus in clinical applications due to its critical role in accurately and efficiently identifying lesions from medical images, thereby supporting diagnosis and treatment planning [5]. Recent years have witnessed significant progress in the development of automated segmentation methods tailored to various medical imaging tasks. In 2015, Ronneberger et al. [6] introduced UNet, which marked a significant advancement in medical image segmentation by leveraging skip connections to preserve spatial detail. This architecture has since been extensively utilized across various segmentation tasks. In 2018, Hirasawa et al. [7] introduced CNN as an effective tool for automating the detection of gastric cancer in endoscopic images. In 2020, Zhang et al. [8] refined the UNet architecture by introducing SERes and DAGC blocks in place of conventional convolution and pooling operations, aiming to strengthen the integration of low‐ and high‐level semantic features. In 2021, Wang et al. [9] proposed a multi‐scale input segmentation technique built upon DeepLabV3+, specifically designed to enhance the detection of gastric cancer lesions in histopathological images. Isensee et al. [10] proposed nnUNet, an automated framework that eliminates the dependency on manual parameter adjustments in biomedical image segmentation by automatically configuring network architecture, training strategy, and data preprocessing. In 2023, He et al. [11] proposed a dual‐branch hybrid network combining Swin‐Transformer and UNet for image segmentation of gastric cancer, which effectively aggregates deep feature information and accurately localizes lesion regions. Shaharabany et al. [12] proposed AutoSAM, an enhanced framework that adapts the Segment Anything Model to medical images by overloading its prompt encoder, enabling fully automatic segmentation without manual prompts. Zhang and Liu [13] proposed SAMed, a customized adaptation of the Segment Anything Model for medical image segmentation, which fine‐tunes both the image encoder and mask decoder to better capture domain‐specific features. In 2024, Zhang et al. [14] proposed an improved Mask R‐CNN model for the detection and lesion segmentation of early gastric cancer, demonstrating superior performance in both tasks. Haq et al. [15] proposed an improved GoogLeNet and ViT model combined with the Faster R‐CNN method to realize multi‐class classification of endoscopic images and regional recognition and segmentation of gastric cancer, yielding superior performance. Chen et al. [16] proposed MA‐SAM, a modality‐agnostic adaptation of the Segment Anything Model for 3D medical image segmentation, which introduces a modality‐independent prompt encoder and a SAM‐friendly 3D feature extractor. Their approaches all demonstrated satisfactory performance in gastric cancer segmentation.

Despite their demonstrated effectiveness, deep learning‐based segmentation models are constrained by several intrinsic limitations. Firstly, their segmentation accuracy has yet to reach an ideal level, making them insufficient for practical applications. Secondly, their complex architectures and large parameter sizes result in high computational overhead and prolonged inference time, rendering them unsuitable for deployment in resource‐constrained environments such as embedded systems. Furthermore, their generalization capability is limited; while they perform well on training datasets, their performance degrades significantly on external datasets. Additionally, networks that simultaneously achieve low parameter count, high accuracy, and strong generalization ability remain scarce. Therefore, there is an urgent need for a lightweight, high‐accuracy, and well‐generalized network to meet real‐world application demands.

To tackle these challenges, we introduce a novel gastric cancer pathological image segmentation model built upon the Segment Anything Model (SAM) [17], termed GC‐SAM (Segment Anything Model in Gastric Cancer). Our method employs a lightweight image encoder to effectively capture tumor morphological characteristics, while improving segmentation accuracy through fine‐tuning of the prompt encoder and mask decoder. The key contributions of this work are summarized as follows:

1. We successfully adapted the widely recognized and robust SAM model for the segmentation of gastric cancer pathological tissue images.
2. Fine‐tuning of the prompt encoder and mask decoder is introduced into the SAM model, achieving superior results on different internal and external gastric cancer datasets.
3. Knowledge distillation is applied to the image encoder within the SAM model, facilitating its deployment on resource‐constrained embedded medical devices.

## 2 Methods

### 2.1 Motivation

Kirillov et al. [17] presented SAM, a cutting‐edge segmentation model that has been trained on a dataset of over 1 billion masks from 11 million natural images. Based on the Vision Transformer (ViT) [18], SAM achieves remarkable results on real images and enables zero‐shot object segmentation, making retraining unnecessary. SAM is unique because it can create segmentation masks with various prompts, such as bounding boxes and points, allowing semantic object suggestions at the pixel level and positional object indications at the region level. Research [19, 20] indicates that SAM exhibits high adaptability and achieves robust results on various segmentation challenges.

Despite SAM's success, significant differences between medical and natural images pose challenges for its direct application. Medical images often exhibit low contrast, intricate structural details, and variability due to observer subjectivity. Moreover, multiple imaging modalities and diverse anatomical objects with distinct characteristics further complicate segmentation tasks [21]. These factors collectively constrain SAM's zero‐shot learning capabilities in the medical domain. Additionally, the original SAM's image encoder, based on ViT‐h, comprises over 600 million parameters. Its substantial computational demands and high resource consumption restrict its deployment in resource‐constrained embedded medical devices.

**FIGURE 1:**
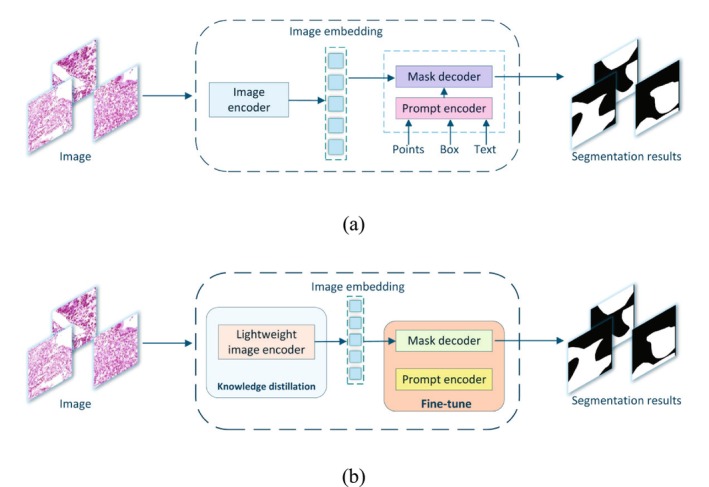
The overall architecture of the system. (a) The structure of the SAM model. (b) The structure of the GC‐SAM model.

This work presents GC‐SAM, a segmentation model tailored for gastric cancer pathology images, based on the SAM framework, as illustrated in Figure 1b. The model consists of three primary components: a lightweight image encoder, a prompt encoder, and a mask decoder. First, the lightweight image encoder processes the input image, which generates feature maps that are then passed to the image embedding. Next, the embedded image is fed into the fine‐tuned mask decoder to generate the corresponding segmentation mask. Moreover, the prompt encoder has been fine‐tuned to effectively utilize bounding box information during data processing.

### 2.2 Segment Anything Model

Figure 1a delineates the three principal modules of the SAM: the image encoder, the prompt encoder, and the mask decoder. The image encoder, architecturally analogous to the Vision Transformer (ViT), employs a masked autoencoder (MAE) [22] for self‐supervised pre‐training and represents the most computationally demanding component within the SAM framework. To accommodate varying computational budgets, SAM offers three pre‐trained weight configurations for the image encoder: ViT‐h, ViT‐l, and ViT‐b. The prompt encoder is responsible for encoding and embedding diverse forms of user input, including spatial cues such as points and bounding boxes, thereby facilitating effective feature representation for downstream mask decoding. The mask decoder is designed to be lightweight, processing image embeddings and prompt embeddings to generate the final segmentation results. This architectural design preserves high‐performance segmentation capabilities while reducing computational complexity, making it more efficient for practical applications.

#### 2.2.1 Image Encoder

The image encoder employs a modified version of the MAE, specifically adapted to process input images with resolutions up to 1024 × 1024.

#### 2.2.2 Prompt Encoder

In the prompt encoding framework, SAM distinguishes between two types of prompts: sparse prompts (points, box, and text) and dense prompts (masks). For encoding sparse prompts like points and bounding boxes, SAM integrates positional encoding with learned embeddings [23]. Specifically, points are represented through two learnable tokens, which encode foreground and background information, respectively. In contrast, the encoding of bounding boxes relies on the coordinates of their top‐left and bottom‐right corners. Additionally, free‐form textual input is encoded using a pre‐trained text encoder [24]. For dense mask prompts, which maintain the same spatial resolution as the input image, SAM employs convolutional layers for embedding, followed by an element‐wise summation with the image embeddings.

#### 2.2.3 Mask Decoder

The mask decoder is structured to be computationally efficient, incorporating several critical components: two Transformer layers, a dynamic mask prediction head, and an Intersection‐over‐Union (IoU) score regression head. The mask prediction head generates three masks, each downsampled by a factor of 4. These masks represent the segmentation of the entire object, a partial segment, and specific subregions within the object.

### 2.3 Fine‐Tuning Algorithm

Fine‐tuning [25] represents an essential technique in the domain of deep learning, often classified as a form of transfer learning. Transfer learning aims to enhance model performance on new tasks by utilizing knowledge acquired from related tasks. This process not only accelerates the adaptation of neural networks to new datasets but also minimizes the computational effort and time needed for model retraining.

This research utilized a fine‐tuning strategy, where the image encoder's weights were frozen to prevent further adjustments. By fine‐tuning the prompt encoder and mask decoder, the model demonstrated enhanced capability in identifying previously unseen tumor regions in gastric cancer pathology slides. Compared to feature extraction‐based transfer learning, this fine‐tuning approach resulted in notable improvements in accuracy and precision, while simultaneously reducing computational costs.

### 2.4 Knowledge Distillation

The SAM image encoder, due to its extensive parameterization, demands substantial memory, which can be prohibitive for devices with limited computational and storage capacities. Consequently, deploying such deep, high‐performance models on embedded medical devices becomes impractical. To address this challenge, knowledge distillation was employed in this study to streamline and optimize the image encoder of SAM.

**FIGURE 2:**
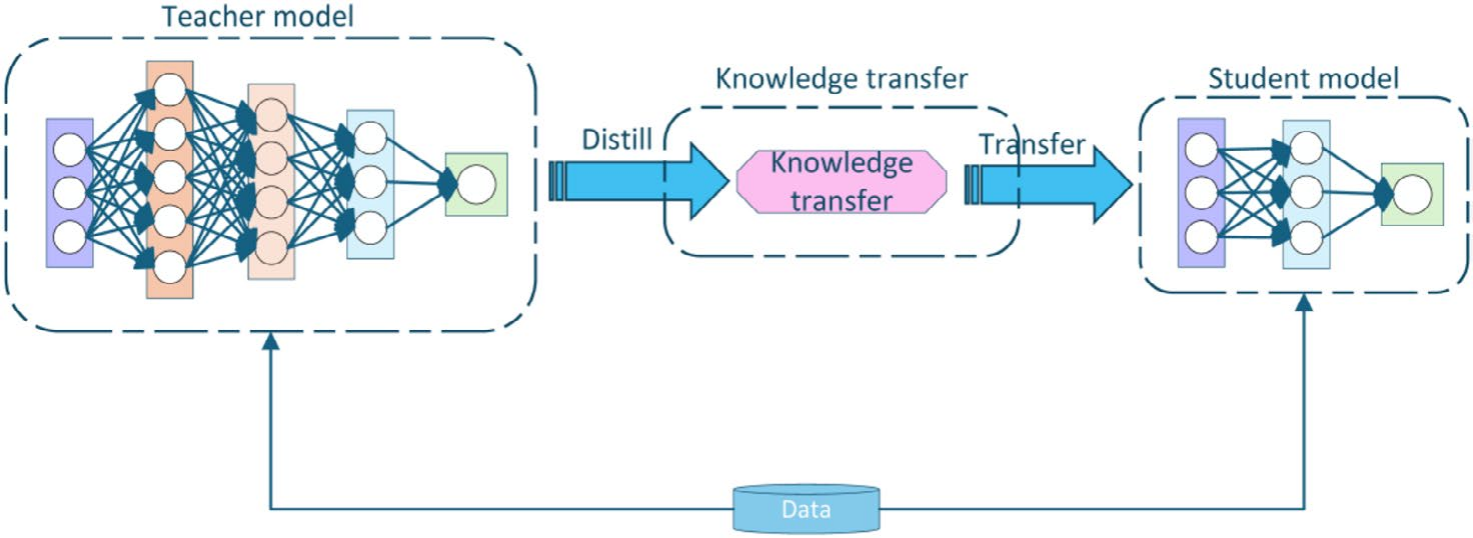
Architecture of knowledge distillation.

Knowledge distillation [26, 27, 28] is widely recognized as an effective model compression technique, enabling the adaptation of complex models to hardware with limited processing capabilities. By mimicking the output distribution of the teacher model, the student model leverages the amplification of differences through softmax to make its output distribution closer to the soft label distribution of the teacher model. This enables the student model to learn the relative relationships between categories, decision boundaries, and confidence information. Knowledge distillation not only achieves model compression but also improves performance, accelerates inference, and enhances the model's generalization ability. This technique results in accelerated inference and reduced memory requirements when models are deployed on mobile or resource‐constrained devices [29]. The architecture of the knowledge distillation model is illustrated in Figure 2.

Let the teacher model *T* produce an output via a softmax $P_{T}=\text{softmax}\left(a_{T}\right)$, where $a_{T}$ represents the weighted sum at the final layer. In contrast, let S denote the parametric student model, with weights $W_{S}$ and output probabilities $P_{S}=\text{softmax}\left(a_{S}\right)$, where $a_{S}$ represents the pre‐softmax output of the student model. During the training phase of the student model, the learning objective involves aligning the student's output $P_{S}$ not only with the ground‐truth labels $y_{\text{true}}$, but also with the soft targets $P_{T}$ produced by the teacher model *T*. To facilitate this alignment, a temperature scaling parameter τ>1 is applied to the teacher's logits, generating softened probability distributions. These softened outputs convey richer relational information among classes, thereby enhancing the effectiveness of knowledge transfer. To ensure consistency in the comparison process, the same temperature parameter τ is applied to the logits of both the student $\left(P_{S}^{\tau }\right)$ and teacher models $\left(P_{T}^{\tau }\right)$. The temperature‐scaled outputs of the two models are defined as follows:

(1) $$P_{T}^{\tau }=\text{softmax}\left(\frac{a_{T}}{\tau }\right)$$

(2) $$P_{S}^{\tau }=\text{softmax}\left(\frac{a_{S}}{\tau }\right)$$

Subsequently, the student model is optimized by minimizing the following objective function:

(3) $$L_{\mathrm{KD}}\left(W_{s}\right)=H\left(y_{\text{true}},P_{s}\right)+\lambda H\left(P_{T}^{\tau },P_{S}^{\tau }\right)$$

In Equation (3), H denotes the standard cross‐entropy function, while λ is a hyperparameter that regulates the trade‐off between two loss components. The first component measures the discrepancy between the student model's predictions and the ground‐truth labels. The second component encourages the student model to approximate the softened output distribution produced by the teacher model.

In this study, we adopted the training strategy of SAM, fine‐tuned the teacher model ViT‐b using the SAM SA‐1B dataset to generate a smaller student model (Vision Transformer small, ViT‐s). This approach significantly reduces the model's complexity and computational resource requirements, leading to the development of a lightweight model. By leveraging knowledge distillation, the student model retains much of the performance of the teacher model while minimizing computational and storage overhead, making it suitable for deployment on resource‐constrained devices.

## 3 Experiments

### 3.1 Datasets and Evaluation Metrics

In this work, gastric cancer histopathological images were sourced from four prominent competitions in the domains of big data and artificial intelligence. These datasets served as the foundation for training and evaluating the proposed GC‐SAM model.

**BOT**: The 2017 China Big Data Artificial Intelligence Innovation and Entrepreneurship Competition (Brain of Things, BOT) Pathological Section Recognition AI Challenge provided by a number of pathologists personally marked 700 gastric cancer digital pathological samples. In these accurately marked samples, we selected 560 pathological section samples with gastric cancer lesions for this study.

**SEED1**: 732 digital pathological section samples with gastric cancer lesions with fine marks provided by the Cancer Risk Intelligent Diagnosis Track of the 2020 “Hualu Cup” SEED Jiangsu Big Data Development and Application Competition.

**SEED2**: 2021 “SEED” the second Jiangsu Big Data Development and Application Competition (SEED2) Medical and health track provided 1770 digital pathological section samples with gastric cancer lesions.

**TCGA**: 60 histopathological images of gastric cancer from the cancer Genome Atlas (TCGA) [30].

Data provided by different sources, BOT, SEED1, and SEED2 constituted the internal dataset for this study, including 2427 gastric cancer histopathological images. To fully evaluate the generalization power and reliability of GC‐SAM, In this experiment, we utilized a diverse external validation dataset comprising 60 gastric cancer histopathological images from multi‐ethnic patients worldwide, sourced from TCGA. These pathological sections were captured using standardized Hematoxylin and Eosin (H&E) staining techniques at a magnification of 20, with images represented in RGB three‐channel format. The annotations were meticulously performed by physicians from Sun Yat‐sen University. To preprocess this dataset, we employed a region‐overlapping cropping method, which involved dividing the images into multiple overlapping patches. This approach not only effectively mitigates edge effects but also expands the gastric cancer histopathological image dataset, thereby reducing the risk of overfitting to a certain extent. Through this external verification, we can check whether GC‐SAM can maintain stable and accurate performance on data from different sources, so as to further confirm the effectiveness and practical value of GC‐SAM model. Additionally, the performance of GC‐SAM in external validation will also provide valuable insights to guide us to optimize the performance of the model in future studies.

**FIGURE 3:**
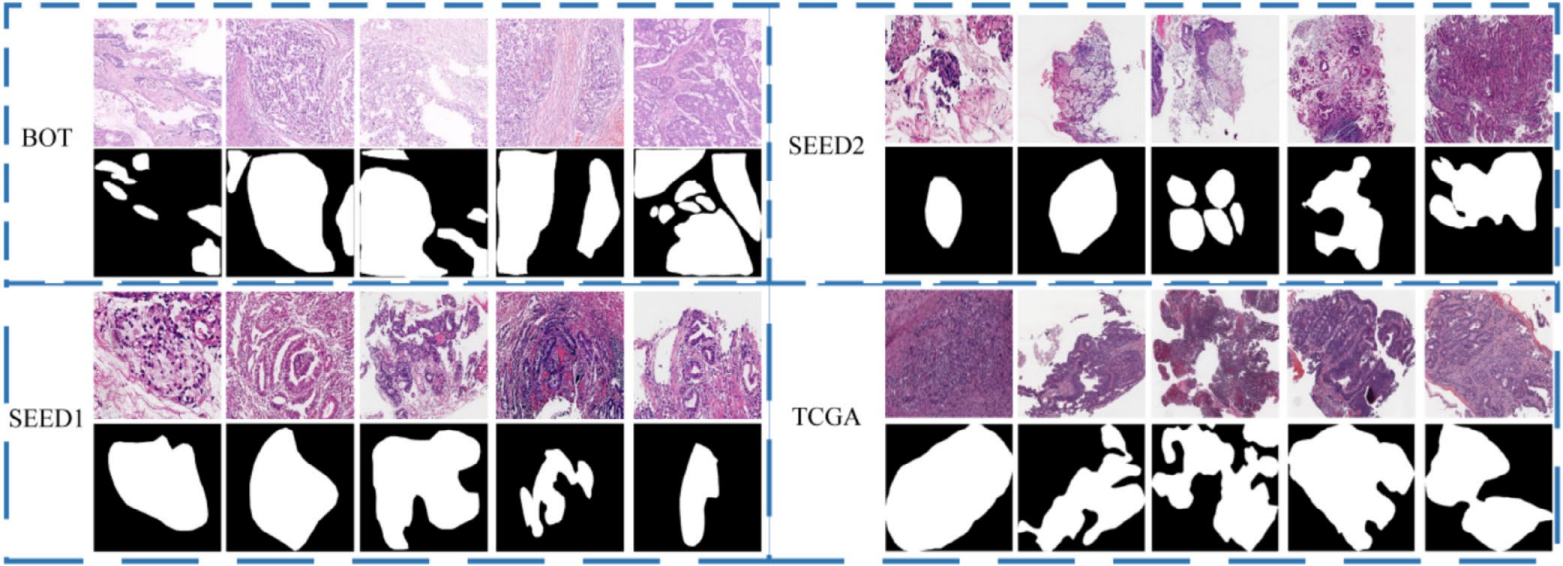
Samples of gastric cancer images from four datasets. White pixels represent the tumor area, while black pixels represent the normal area.

Sample images from the four datasets used in this study are illustrated in Figure 3.

Table 1 more intuitively presents key information about the internal and external datasets used in this study, including their source, quantity, size, and data type.

**TABLE 1:** Key information of the datasets used.

	Dataset	Source	Quantity	Scale	Type
Internal dataset	BOT (2017)	The 2017 China Big Data Artificial Intelligence Innovation and Entrepreneurship Competition	560	2048 × 2048	.tiff
	SEED1 (2020)	The 2020 “HuaLu Cup”	732	2048 × 2048	.png
	SEED2 (2021)	The 2021 “HuaLu Cup”	1770	2048 × 2048	.png
External database	TCGA	The Cancer Genome Atlas Program	60	102,400 × 102,400	.svs

To comprehensively evaluate the performance of the model, this study used the Dice coefficient (Dice), mean intersection over union (mIoU) and pixel accuracy (Pixel Acc) as evaluation metrics to assess the similarity between the ground truth annotations and the segmentation results produced by the model.

(4) $$\text{Dice}=\frac{2\times \mathrm{TP}}{\left(\mathrm{TP}+2\times \mathrm{TP}+\mathrm{FN}\right)}$$

(5) $$\text{mIoU}=\frac{1}{k+1}\sum_{i=0}^{k}\frac{{\mathrm{TP}}_{i}}{{\mathrm{TP}}_{i}+{\mathrm{FP}}_{i}+{\mathrm{FP}}_{i}}$$

(6) $$\text{Pixel}\;\mathrm{Acc}=\frac{\sum_{i=0}^{k}p_{\mathrm{ii}}}{\sum_{i=0}^{k}\sum_{j=0}^{k}p_{\mathrm{ij}}}$$

TP represents true positive, where both the prediction and actual sample are positive. TN represents true negative, where both the prediction and actual sample are negative. FP represents the number of samples that are negative but predicted as positive, while FN represents the number of samples that are positive but predicted as negative. The term $\sum_{i=0}^{k}p_{\mathrm{ii}}$ represents the sum of all correctly predicted pixels, and $\sum_{i=0}^{k}\sum_{j=0}^{k}p_{\mathrm{ij}}$ represents the total number of pixels in the image.

### 3.2 Implementation Details

#### 3.2.1 Color Correction

**FIGURE 4:**
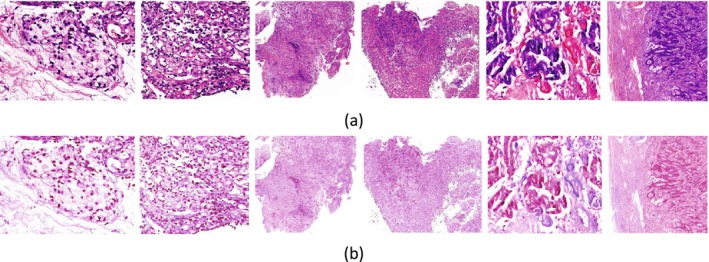
Images before and after color correction. (a) Original image. (b) Color‐corrected image.

The datasets used in this study were obtained from different platforms. As shown in Figure 4, color shifts may occur during image collection or scanning of gastric cancer tissue pathology samples due to differences in brightness and color response characteristics across various platforms. To address these color shifts and enhance the accuracy while maintaining uniformity among various gastric cancer tissue pathology images, a color correction technique [31] was used. Figure 4 presents the original images and the images after color correction. The color consistency has been evidently improved.

#### 3.2.2 Experiment Settings

To support model development and performance assessment, the internal dataset was partitioned into three distinct subsets: 60% for training, 20% for validation, and 20% for testing. This stratification ensures a balanced distribution of samples, allowing the model to adequately learn representative patterns while preventing overfitting. For the optimization objective, Dice Loss, a metric commonly adopted in medical image segmentation, was utilized due to its effectiveness in handling class imbalance and its demonstrated robustness across diverse segmentation scenarios. The network hyperparameter settings are shown in Table 2.

**TABLE 2:** Network hyperparameter settings.

Name	Parameter size
Optimizer	AdamW
Epoch	50
Batch_size	16
Learning_rate	0.001
Weight_decay	4e^−6^

### 3.3 Comparative Experiment

#### 3.3.1 GC‐SAM Versus SAM

To demonstrate the necessity of fine‐tuning and knowledge distillation in enhancing model performance, we conducted a comparison experiment of GC‐SAM and SAM.

Table 3 summarizes Dice, mIoU, Pixel Acc, speed, and parameter count, which are given to evaluate the accuracy and complexity of the model, respectively. Compared with SAM, GC‐SAM has achieved significant improvement in each evaluation index. Specifically, GC‐SAM improves the Dice coefficient by 5.9%, indicating more accurate segmentation. The mIoU shows a substantial rise of 79.6%, reflecting better overlap and boundary precision. Pixel Accuracy is enhanced by 8.6%, reaching 0.7921. In addition, by employing fine‐tuning and knowledge distillation techniques, GC‐SAM achieves an 83.6% reduction in inference time, running at 1.29 s compared to SAM's 7.87 s. Last but not least, the model size is drastically reduced by 89.2%, with parameters dropping from 93.73 to just 10.13 M. These results highlight the feasibility of deploying GC‐SAM on embedded medical devices.

**TABLE 3:** Segmentation results of GC‐SAM and SAM on the internal validation dataset.

Method	Dice	mIoU	Pixel Acc	Speed (s)	Parameters (M)
SAM	0.7729	0.3621	0.7291	7.87	93.73
GC‐SAM	0.8186	0.6504	0.7921	1.29	10.13
Improvement	**+5.9%**	**+79.6%**	**+8.6%**	**−83.6%**	**−89.2%**

*Note:* The “Improvement” quantitatively illustrates the gains achieved by GC‐SAM over the baseline SAM model. The green is used for highlighting purpose only.

#### 3.3.2 GC‐SAM Versus Other Segmentation Networks

To comprehensively evaluate the performance of the proposed GC‐SAM model, the compared segmentation networks are categorized into three groups. The first group includes classical segmentation networks (FCN [32], SegNet [33], ContextNet [34], CGNet [35] and UNet [6]), which have played a foundational role in early semantic segmentation research. The second group consists of advanced segmentation methods (PSPNet [36], the DeepLab series [37, 38] and nnUNet [10]), which have demonstrated outstanding performance across a variety of segmentation tasks. The third group comprises SAM‐based models and their variants (AutoSAM [12], SAMed [13] and MA‐SAM [16]), which are developed based on the Segment Anything architecture and adapted specifically for medical imaging scenarios. A systematic comparison with these three categories of models validates the effectiveness and advancement of GC‐SAM in medical image segmentation tasks.

The results are summarized in Table 4. It is worth noting that GC‐SAM achieved significant results on all comparison indicators. Specifically, GC‐SAM achieves a Dice of 0.8186, surpassing segmentation networks such as DeepLabV3+ (0.7796) and nnUNet (0.7705), which demonstrates its enhanced ability to capture complex features in medical images. Although MA‐SAM and DeepLabV3 achieve comparable or slightly higher mIoU, GC‐SAM attains similar performance with a significantly smaller model size of only 10.13M parameters, representing a reduction of approximately five to eleven times compared to DeepLabV3+ (58.75M) and MA‐SAM (115.27M). This substantial compression leads to significantly lower computational costs and faster inference, both of which are essential for real‐time medical applications. In addition, GC‐SAM achieves a Pixel Acc of 0.7912, outperforming all compared methods, including AutoSAM, SAMed, and several widely‐used classical segmentation networks. In summary, GC‐SAM not only excels in segmentation accuracy compared to top‐tier classical and SAM‐based models but also offers remarkable improvements in efficiency. This enables real‐time image analysis applications of GC‐SAM in scenarios such as disease screening, telemedicine, and edge computing.

**TABLE 4:** Quantitative comparison between other segmentation networks and GC‐SAM.

Method	Dice	mIoU	Pixel Acc	Parameters(M)
FCN	0.6013	0.4656	0.6263	18.16
SegNet	0.6833	0.5233	0.5790	29.44
ContextNet	0.7315	0.5992	0.7219	112
CGNet	0.7696	0.6483	0.7596	0.491
PSPNet	0.7691	0.6466	0.7400	48.63
UNet	0.6939	0.5503	0.6539	31.04
DeepLabV3	0.7717	0.6541	0.7625	58.63
DeepLabV3+	0.7796	0.6003	0.7680	58.75
nnUNet	0.7705	0.6588	0.7553	46.33
AutoSAM	0.7395	0.6051	0.7309	93.74
SAMed	0.7075	0.5814	0.6777	90.46
MA‐SAM	0.7681	0.6592	0.7603	115.27
GC‐SAM	0.8186	0.6504	0.7912	10.13

*Note:* 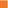: Best, 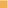: Second‐best, 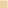: Third‐best.

### 3.4 External Validation

**FIGURE 5:**
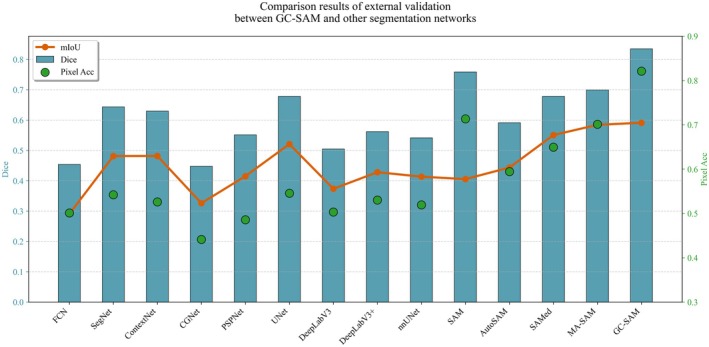
Comparison results of external validation between GC‐SAM and other segmentation networks.

To evaluate the generalization ability of the GC‐SAM model, especially its zero‐shot transfer potential, experiments were conducted on the external TCGA dataset, which was not involved during training. As the model had no prior exposure to this dataset, the assessment enables a robust examination of its capacity to generalize to previously unseen pathological samples. Figure 5 illustrates the quantitative performance comparison between GC‐SAM and representative models from the three segmentation categories. GC‐SAM demonstrates outstanding performance across all evaluation metrics, particularly excelling in the Dice coefficient, where it achieves a remarkable score of 0.835, significantly surpassing other models. This indicates that GC‐SAM holds a distinct advantage in the precision of image segmentation, enabling more accurate delineation between foreground and background regions. Furthermore, GC‐SAM's superior performance in mIoU underscores its enhanced capability in handling tasks involving class overlap and boundary recognition. Additionally, GC‐SAM's Pixel Acc of 0.8210 is another strong point, reflecting its high classification accuracy at the pixel level. In summary, the comprehensive superiority of GC‐SAM on the external validation set, particularly in key metrics such as Dice and Pixel Acc, compared with other segmentation networks, showcases its robust capabilities in complex image segmentation tasks.

## 4 Discussion

### 4.1 Comparison With Existing Models

GC‐SAM demonstrates superior performance compared to conventional convolutional neural network (CNN)‐based models such as FCN and UNet, as it is more effective in capturing the irregular and complex structures present in gastric cancer tissues. This advantage can be attributed to the high‐capacity image encoder inherited from the SAM framework. In addition, GC‐SAM surpasses more advanced segmentation models, including DeepLabV3+, nnUNet, and PSPNet, on both internal and external datasets, indicating its strong generalization capability.

### 4.2 SAM‐Based Segmentation in Medical Imaging

Recent research efforts have explored the adaptation of SAM for medical image segmentation, as systematically reviewed by Zhang et al. [39]. However, most existing approaches, such as AutoSAM, SAMed, and MA‐SAM, involve only partial fine‐tuning of the model components or retain high computational complexity. In contrast, the proposed GC‐SAM performs joint fine‐tuning of the prompt encoder and mask decoder, and incorporates knowledge distillation to compress the image encoder. As a result, GC‐SAM achieves the best segmentation performance while significantly reducing the model size to 10.13 M.

### 4.3 Limitations and Future Work

This study primarily focuses on gastric cancer histopathological images, and the adaptability of GC‐SAM to other pathological types and cancer varieties requires further validation. Moreover, the current prompting strategy mainly relies on image‐region‐based inputs and does not fully exploit multimodal information. Future work will explore integrating natural language descriptions or structured textual prompts to enable richer multimodal interactions.

## 5 Conclusion

This study proposed GC‐SAM, a lightweight segmentation network specifically designed for gastric cancer segmentation. GC‐SAM improves the encoder and decoder components, effectively replacing traditional, computationally intensive network components. To comprehensively evaluate the performance of GC‐SAM, we compared it with representative segmentation models from three categories: classical networks, advanced methods, and SAM‐based variants. Evaluations were conducted on both internal and external validation datasets.

1. Precision: GC‐SAM demonstrates significant improvements in precision, outperforming the networks mentioned above in terms of Dice, while maintaining a compact model size and low computational overhead.
2. Efficiency and speed: GC‐SAM demonstrates significant improvements in efficiency and speed compared to the original SAM model. The parameter count and the processing speed are reduced by 89.2% and 83.6%, respectively.
3. Generalization: GC‐SAM demonstrates robust generalization capabilities, as shown by experiments on the external validation dataset.

In summary, the proposed GC‐SAM offers a precise and efficient solution for gastric cancer segmentation, providing a promising pathway for the deployment of deep learning models in embedded medical devices.

## Author Contributions

Project administration and funding acquisition: Juan Li and Dabiao Wang. Conceptualization, methodology, validation, writing – original draft: Lanlan Li, Chongyang Wang, and Yi Geng. Formal analysis: Tao Chen and Ziyue Wang. Data curation: Kaixin Lin. Writing – review and editing: Hongan Wei, Jianping Wang, and Decao Niu.

## Ethics Statement

This study used publicly available and anonymized datasets (SEED, BOT, and TCGA) and did not involve new human subject experiments or collection of identifiable private data. Therefore, ethical approval and informed consent were not required according to national regulations (Article 32 of China's Ethical Review Measures and Article 73 of the Personal Information Protection Law).

## Conflicts of Interest

The authors declare no conflicts of interest.

## Data Availability

The public datasets that support the findings of this study are available from the following sources: The Cancer Genome Atlas (TCGA) database. The SEED dataset, released through the 2020 and 2021 “Hualu Cup” SEED Jiangsu Big Data Development and Application Competitions. The BOT dataset, released through the 2017 China AI Innovation and Entrepreneurship Competition.
